# TD-YOLO: Tomato Leaf Disease Detection Based on Hypergraph Multi-Scale Aggregation

**DOI:** 10.3390/s26196340

**Published:** 2026-10-08

**Authors:** Yuwen Liu, Xi Feng, Mengting Liu, Shunxin Cao

**Affiliations:** 1 Base for Innovative Methods Promotion Application and Demonstration of Anhui Province, Anhui University of Science and Technology, Huainan 232001, China; lyw010@aust.edu.cn; 2 School of Artificial Intelligence, Anhui University of Science and Technology, Hefei 231131, China; 3 School of Electrical and Information Engineering, Anhui University of Science and Technology, Huainan 232001, China; xfeng@aust.edu.cn; 4 Faculty of Modern Health Care, Anhui Sanlian University, Hefei 230601, China; mtliu112358@163.com

**Keywords:** tomato leaves, disease detection, TD-YOLO, lightweight model, model adaptability

## Abstract

To address the challenges of tomato leaf diseases, including significant variations in disease size, discrete spatial distribution in complex field environments, and the difficulty in distinguishing early symptoms of certain diseases, which lead to low detection accuracy and a large number of model parameters, this paper proposes a tomato leaf disease detection model, TD-YOLO, based on multi-scale hypergraph aggregation. During the feature extraction stage, the RetC3k2 is employed to enhance the shallow network’s ability to detect disease regions. The C2TSSA attention mechanism is introduced to filter out key diseases and suppress background interference. At the same time, the GDown adaptive downsampling module is designed to reduce the model size while preserving feature integrity. In the neck network section, a multi-scale hypergraph aggregation structure is constructed by integrating a multi-scale semantic aggregation layer, the HyperConv, and a MANet network. By connecting disease features at different spatial locations through hyperedges, the network models high-order associative relationships and achieves a refined representation of multi-scale features. Experimental results show that on the Tomato-Village-Diseases dataset, TD-YOLO achieves an mAP50 of 92.5%, a 7.1% improvement over the baseline YOLOv11, with only 2.21M parameters and a computational cost of 6.8 GFLOPs. On the Tomato-Leaf-2-v1 dataset, the mAP50 reached 81.0%, outperforming all comparison models and demonstrating the model’s strong adaptability. This study provides an efficient and feasible technical solution for the accurate detection of tomato leaf diseases.

## 1. Introduction

Tomatoes are one of the most important cash crops worldwide, ranking among the top vegetable crops in terms of cultivated area and economic output. However, during their growth, tomatoes are susceptible to various diseases, including nutrient deficiencies, tomato spotted wilt virus, early blight, and late blight, which severely impact yield and fruit quality and pose a persistent threat to sustainable agricultural development [1]. Traditional disease diagnosis methods rely heavily on manual visual assessment by experts, which is not only time-consuming and labor-intensive but also highly subjective, making it difficult to meet the urgent demand for real-time, automated detection in modern precision agriculture [2,3,4].

In recent years, computer vision technologies represented by deep learning have achieved breakthrough progress in the field of plant disease recognition [5]. Object detection algorithms, as one of the core technologies in this field, can be divided into two categories based on their network architecture: Two-stage detectors represented by R-CNN and Faster R-CNN [6,7] achieve high detection accuracy, yet suffer from heavy computational overhead and slow inference speed, making it difficult to meet the requirements of real-time applications. Single-stage detectors represented by the YOLO series [8,9], SSD [10], and RT-DETR [11], achieve a better balance between accuracy and speed through end-to-end network design and have become the mainstream solutions for agricultural disease detection.

In the field of tomato disease detection, researchers have conducted extensive work focused on balancing detection accuracy and computational efficiency. In terms of Transformer technology, Abulizi et al. [12] combined Deformable Attention with the AIFI module and adopted FasterNet as the backbone network to propose the FNE-RTDETR model, which significantly reduced computational overhead while maintaining 91.5% accuracy. Bian et al. [13] proposed an improved RT-DETR method based on multi-scale feature fusion for tomato disease detection in complex backgrounds, effectively enhancing the model’s robustness in field scenarios. Tang et al. [14] have carried out research on detection and segmentation and proposed the ConTriNet network [15], which combines three streams of information and uses a ‘divide and conquer’ approach for RGB-thermal saliency detection. Even when some of the data is missing, it can still perform reliably in complex scenes. In terms of YOLO technology, Ramos et al. [16] conducted a systematic comparative analysis of YOLO architectures using 14,368 images, verifying that YOLOv11 offers superior overall performance in terms of accuracy, training time, and inference latency. Improvements to the YOLO framework can be categorized into two directions: regarding accuracy enhancement, Meng et al. [17] integrated the EfficientMSF, C2CU, and CAFMfusion modules to improve YOLOv11n, achieving an mAP50 of 76.5% on a self-built nine-class dataset. Tang et al. [18] proposed the YOLOv11-AIU model, which achieved an mAP50 of 94.1% for severity classification of early blight in tomatoes by employing C3k2_iAFF attention fusion and a Unified-IoU loss function. Wang et al. [19] built the YOLO-SPDNet model by integrating SEAM semantic enhancement and the MLCA lightweight attention mechanism with YOLOv11, achieving an mAP50 of 90.6% in complex natural scenes. In terms of lightweight design and edge deployment, Han et al. [20] proposed the DFR-YOLOv12n model, which achieves synergistic optimization of accuracy, lightweight design, and edge deployment by using SPDConv to preserve fine-grained features, A2C2f_DEConv to enhance texture representation, and MPDIoU to optimize bounding box regression. To enable accurate and timely diagnosis of tomato diseases on edge devices, Zarboubi et al. [21] proposed the CustomBottleneck-VGGNet model and integrated transfer learning, successfully deploying it on mobile devices to achieve offline real-time detection with a recognition accuracy of 99.12%.

The aforementioned studies have advanced the development of intelligent tomato disease detection technology, but there are still shortcomings. First, model lightweighting comes at the expense of detection accuracy, making it difficult to balance accuracy and efficiency. Second, lesions of nutrient deficiency disorders and viral diseases vary drastically in scale and are discretely distributed on leaf blades. Conventional convolutional architectures struggle to capture the spatial correlations and global dependencies between scattered diseases. Third, the adaptability of existing models remains to be verified, as complex lighting conditions and background variations in real-world field scenarios pose severe challenges to model robustness. To address these issues, this paper proposes TD-YOLO, a tomato leaf disease detection model based on multi-scale hypergraph aggregation, using YOLOv11n as the baseline. The main contributions of this paper are as follows:

(1) During the feature extraction stage, the RetC3k2 is introduced to enhance the shallow network’s ability to detect diseased areas. The C2TSSA attention mechanism is employed to filter out key diseases and suppress background noise. Meanwhile the GDown adaptive downsampling module is designed to reduce the model size while preserving feature integrity.

(2) In the Neck section, we construct a multiscale hypergraph aggregation structure that integrates a multiscale semantic aggregation layer, HyperConv module, and a MANet network. By connecting disease features at different spatial locations via hyperedges, we model higher-order correlations among dispersed diseases and achieve a refined representation of multiscale features.

(3) The model adaptation experiments conducted on the Tomato-Village-Diseases and Tomato-Leaf-2-v1 datasets validated the model’s detection accuracy and lightweight design.

## 2. Materials and Methods

### 2.1. Dataset

**Figure 1:**
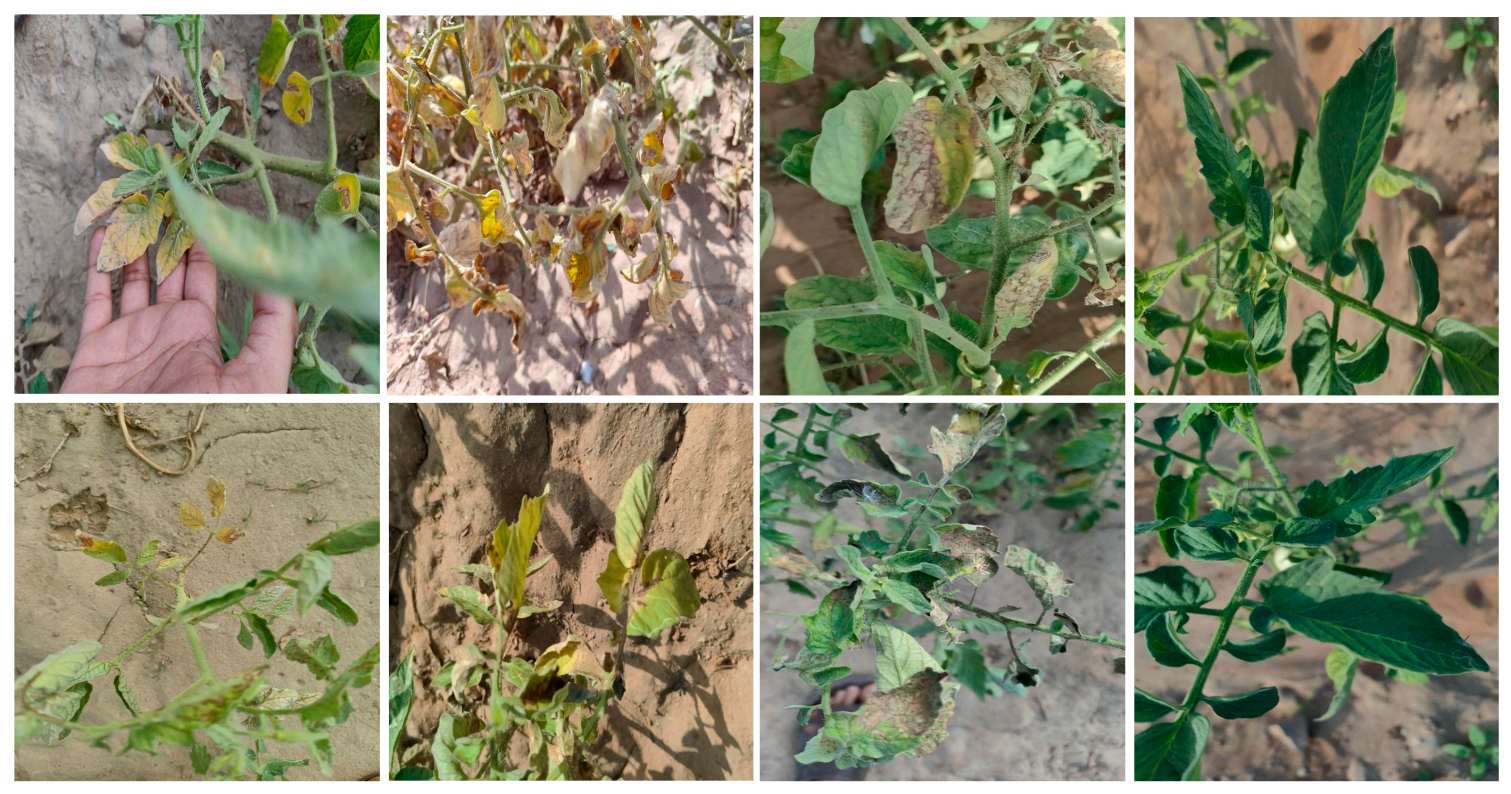
Sample images from the Tomato-Village-Diseases dataset.

The publicly available tomato leaf disease dataset used in this paper, Tomato-Village-Diseases, is an image dataset specifically constructed for detecting tomato leaf diseases in real-world field environments. The images may contain multiple leaves, complex background variations, natural lighting conditions, and varying leaf orientations. all of which are realistic factors that make the dataset more closely resemble actual agricultural scenarios. Meanwhile models trained on this dataset are therefore more valuable for real-world applications. The dataset mainly consists of healthy tomato leaves and three common tomato leaf disorders, including nitrogen deficiency characterized by uniform yellowing and symptoms on old leaves, potassium deficiency with marginal leaf scorch and yellowing, and spotted wilt virus featured by gray-white spots and small black dots. The Tomato-Village-Diseases dataset comprises 4331 tomato leaf images, which are randomly divided into training, validation, and test sets in a 7:2:1 ratio. The dataset underwent perceptual hash-based near-duplicate checking to make sure images of the same scene are only assigned to one subset. Figure 1 shows a partial view of the Tomato-Village-Diseases dataset, featuring images representing each of the four tomato leaf categories.

**Figure 2:**
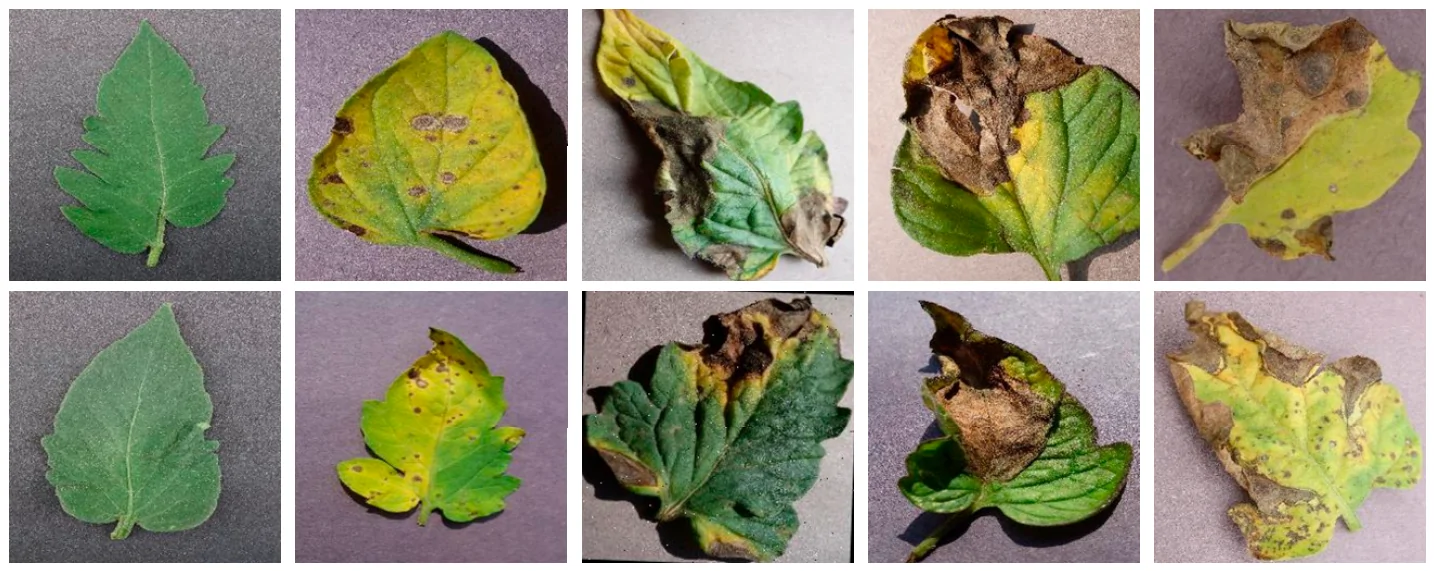
Sample images from the Tomato-Leaf-2-v1 dataset.

In this paper, another dataset named Tomato-Leaf-2-v1 is selected for adaptability experiments for verification. The results not only demonstrate that the TD-YOLO model maintains excellent detection performance for other types of tomato leaf diseases but also highlight the model’s adaptability and robustness. This dataset consists of 8541 images and includes five classes: Early_blight, Late_blight, Leaf_Mold, Septoria_leaf_spot, and Healthy. The dataset was similarly divided into training, validation, and test sets in a 7:2:1 ratio. And the dataset underwent perceptual hash-based near-duplicate checking to make sure images of the same scene are only assigned to one subset. Figure 2 shows a selection of images from the dataset.

### 2.2. TD-YOLO Overall Framework

**Figure 3:**
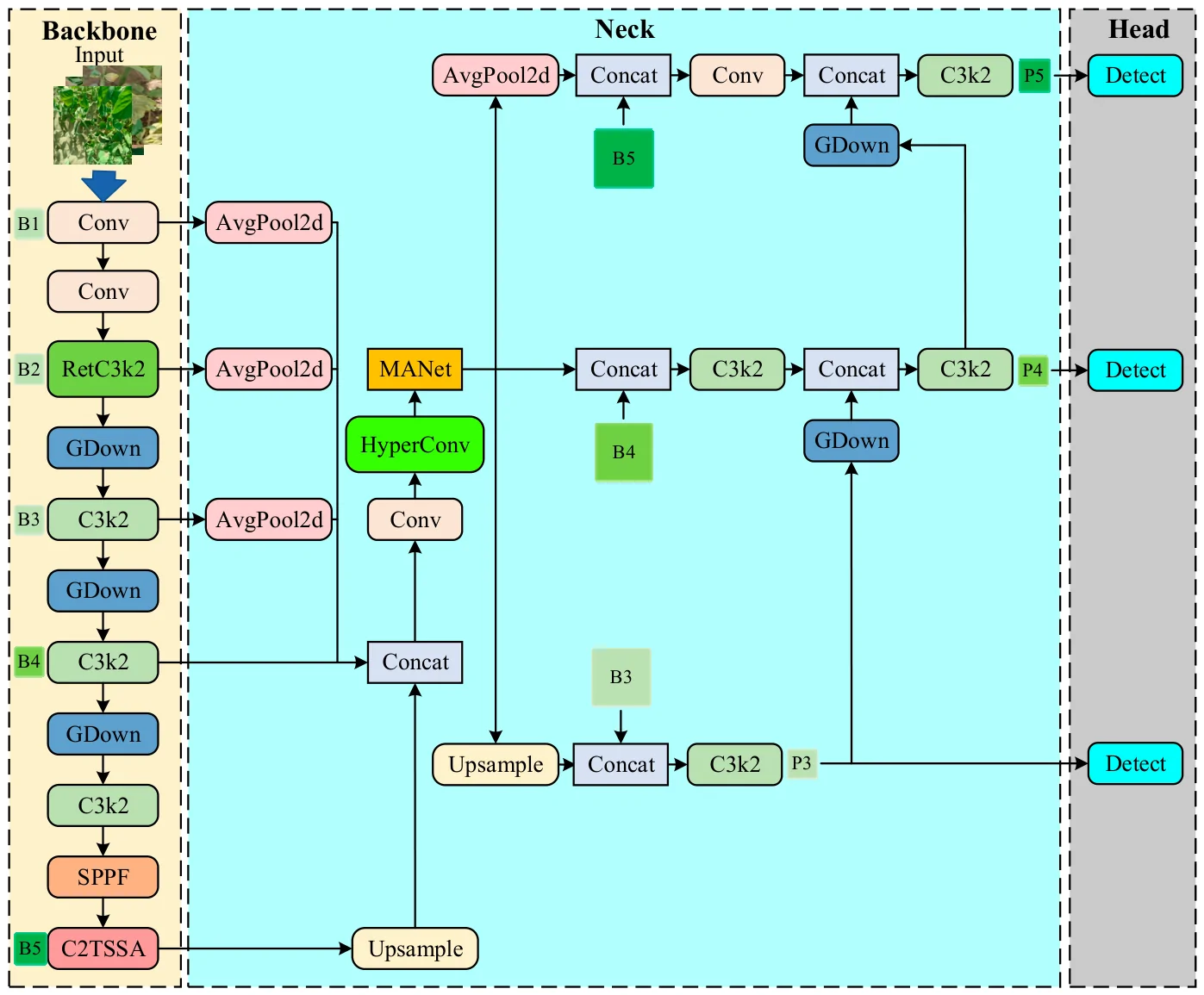
Overall model diagram of TD-YOLOv11.

To address the issues of wide variation in disease sizes, discrete spatial distribution, and high computational complexity of existing models in tomato leaf disease detection, this paper proposes the TD-YOLO detection model, using YOLOv11n as the baseline. The overall framework is shown in Figure 3. In the main network, the RetC3k2 is introduced to enhance shallow-layer disease detection capabilities, while the C2TSSA attention mechanism is used to filter key diseases and effectively suppress background interference. Additionally, the GDown adaptive downsampling module is retained to preserve disease details while reducing feature resolution. In the neck section, a multiscale hypergraph aggregation structure is constructed by integrating a multiscale semantic aggregation layer, the HyperConv module, and the MANet network. By utilizing hyperedge modeling to decompose the higher-order correlation relationships among diseases, this structure achieves a refined representation of multiscale features for tomato leaf diseases.

#### 2.2.1. RetC3k2 Module

To address the challenge that certain lesions in tomato leaf diseases possess tiny sizes, indistinct textures and low contrast against healthy leaf areas, the CSP architecture of the original C3k2 module segments and concatenates input features along channels. While this reduces computational redundancy, its fixed feature interaction mechanism lacks a differentiated response to key diseased areas, causing faint disease signals to be easily overwhelmed by the background during propagation through deeper layers and making them difficult for subsequent detection heads to identify effectively. To address this, this paper introduces a Retention Block [22] based on the first C3k2 module in the backbone network to construct the RetC3k2 module. While retaining the advantages of CSP’s cross-stage connectivity, this approach performs spatial feature recalibration on the feature maps output by the Bottleneck layer.

First, the input feature $X\in R^{C\times H\times W}$ of tomato leaf images is split into two parts along the channel dimension. One part passes through n Bottleneck modules for deep feature extraction, yielding the intermediate feature $F\in R^{C\times H\times W}$. The re-labeling mechanism performs spatial feature re-calibration on F, the calculation process is shown in Equation (1):


(1) $$F_{retoken}=Conv_{1\times 1}(F)+F$$

To enhance the network’s ability to detect diseased areas at an early stage, a dynamic saliency weight allocation strategy is further introduced. The heavily weighted features $F_{retoken}$ are subjected to global average pooling and global max pooling along the channel dimension, producing two feature descriptors of size H×W×1 each. These two descriptors are concatenated along the channel dimension to form a feature map of size H×W×2, and a 1×1 convolution is applied to reduce the channel dimension to 1, followed by a sigmoid activation to generate the spatial saliency map $S_{w}\in R^{1\times H\times W}$, as shown in Equation (2):


(2) $$S_{w}=\sigma (Conv_{1\times 1}(Concat(AvgPool(F_{retoken}),MaxPool(F_{retoken}))))$$

Here, σ represents the sigmoid activation function. Finally, the significance weights are multiplied element-wise with the re-masked features, and the residuals are added to the original Bottleneck output, as shown in Equation (3):


(3) $$F_{out}=F+\alpha \cdot (S_{w}⊙F_{retoken})$$

Here, α represents a learnable scaling factor, and ⊙ denotes element-wise multiplication. Through this process, the network can identify the spatial locations most likely to be diseases at shallow layers, enabling early detection of potential disease-affected areas. This ensures that critical disease information is preserved as it propagates through deeper layers, providing a more reliable feature foundation for subsequent feature selection and hypergraph association modeling.

#### 2.2.2. GDown Sampling and C2TSSA Feature Selection

While enhancements in shallow-layer disease detection have been achieved, deep-layer feature extraction still requires consideration. On the one hand, the resolution of feature maps continuously decreases as the network deepens, and subtle edges and texture information of diseases are easily lost during successive downsampling. Particularly for small-target diseases like spotted wilt virus, information loss exerts a direct negative impact on the recognition accuracy of the detection head. On the other hand, in complex field scenarios, interfering factors such as changes in lighting and soil backgrounds intermingle with disease features, increasing the risk of false positives. The standard convolutional downsampling method used in the last three stages of the original YOLOv11 backbone network, while effectively expanding the receptive field, struggles to balance detail preservation with background suppression. Furthermore, the terminal C2PSA module performs dense attention calculations on all spatial locations, resulting in a significant amount of computational resources being consumed in background areas.

To address the severe loss of detail in conventional downsampling methods, this paper proposes the GDown adaptive downsampling module based on YOLOv9′s [23] ADown. The input feature map is divided along the channel dimension into two parts, $X_{1}$ and $X_{2}$. $X_{1}$ passes through an improved left branch, where standard convolutions are replaced with grouped convolutions to reduce the number of parameters and computational load, supplemented by point-wise convolutions to achieve cross-group channel fusion. Furthermore, residual mappings are introduced to allow shallow-layer disease responses to be directly propagated to deeper layers, thereby preventing the attenuation of weak features. In branch $X_{2}$, average pooling in the right branch is replaced with max pooling to enhance the preservation of high-frequency features such as disease edges and textures. After concatenating the outputs of both branches, channel rearrangement is performed to facilitate information exchange between the different branches. The computational process can be expressed by Equations (4)–(6):


(4) $$X_{left}=Conv_{1\times 1}(Conv_{3\times 3,g}(X_{1})+Conv_{1\times 1,s2}(X_{1}))$$

(5) $$X_{right}=Conv_{1\times 1}(MaxPool_{3\times 3,s2}(X_{2}))$$

(6) $$X_{down}=ChannelShuffle(Concat(X_{left},X_{right}))$$

**Figure 4:**
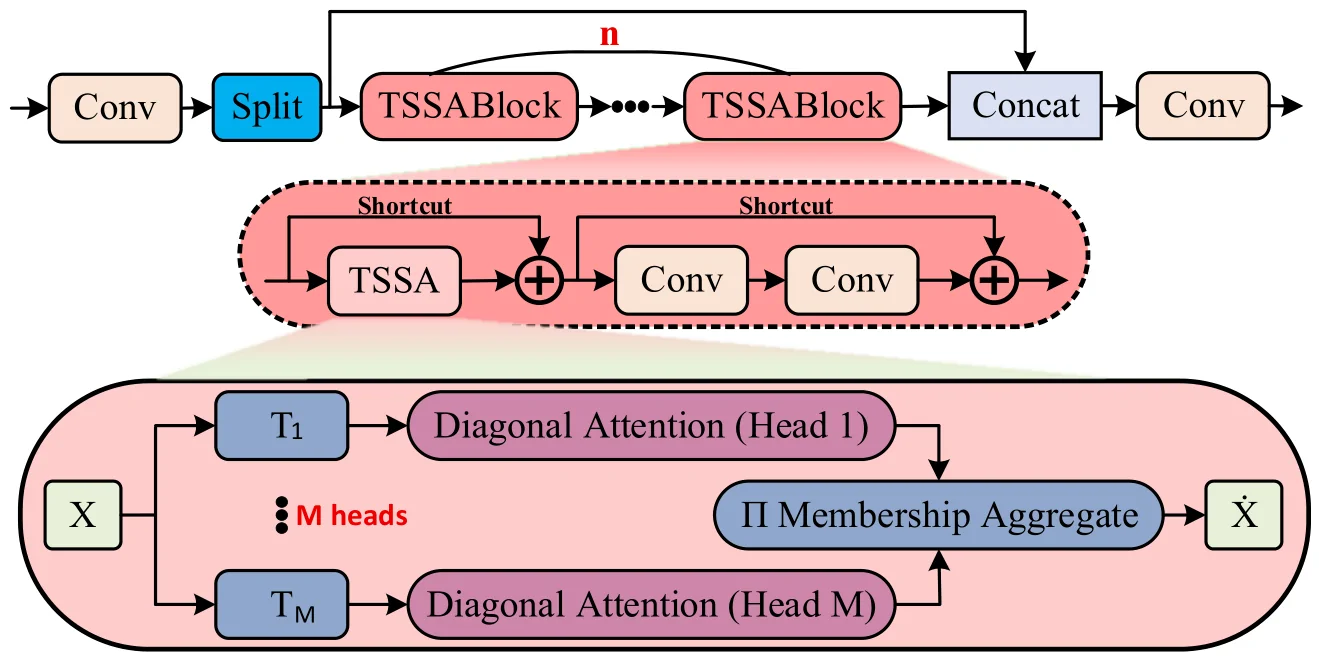
C2TSSA structure diagram.

Here, g represents the number of groups in group convolution, determined by the greatest common divisor of the input and output channels, with an upper limit of 2 to reduce computation while maintaining effective interaction between channels, and s2 denotes a stride of 2. The downsampled feature maps still contain background noise such as leaf veins and lighting effects, requiring further filtering to extract key disease features. The original C2PSA module performs dense attention calculations across all spatial locations, which prevents it from focusing on sparsely distributed key disease features. To address this, this paper introduces the TSSA mechanism [24] to construct the C2TSSA module as a replacement for C2PSA, as shown in Figure 4. First, the input feature $X\in R^{C\times H\times W}$ is grouped along the channel dimension, with each group treated as a feature set $T_{i}\in R^{C_{g}\times H\times W}$, where $C_{g}$ is the number of channels in each group. Subsequently, after calculating the saliency scores for each group, the top M groups with the highest scores are selected for multi-head self-attention, while the remaining groups retain their original features via an identity mapping.

C2TSSA treats channels as feature groups, enabling the model to focus its limited computational resources on disease-related regions and effectively suppress background noise. GDown preserves as much disease detail as possible during the resolution compression process, providing C2TSSA with feature maps that contain complete disease responses. By balancing detail preservation with key feature selection, the model remains lightweight while effectively improving the detection accuracy and robustness of tomato leaf diseases in complex field environments.

#### 2.2.3. Multi-Scale Hypergraph Aggregation Structure

**Figure 5:**
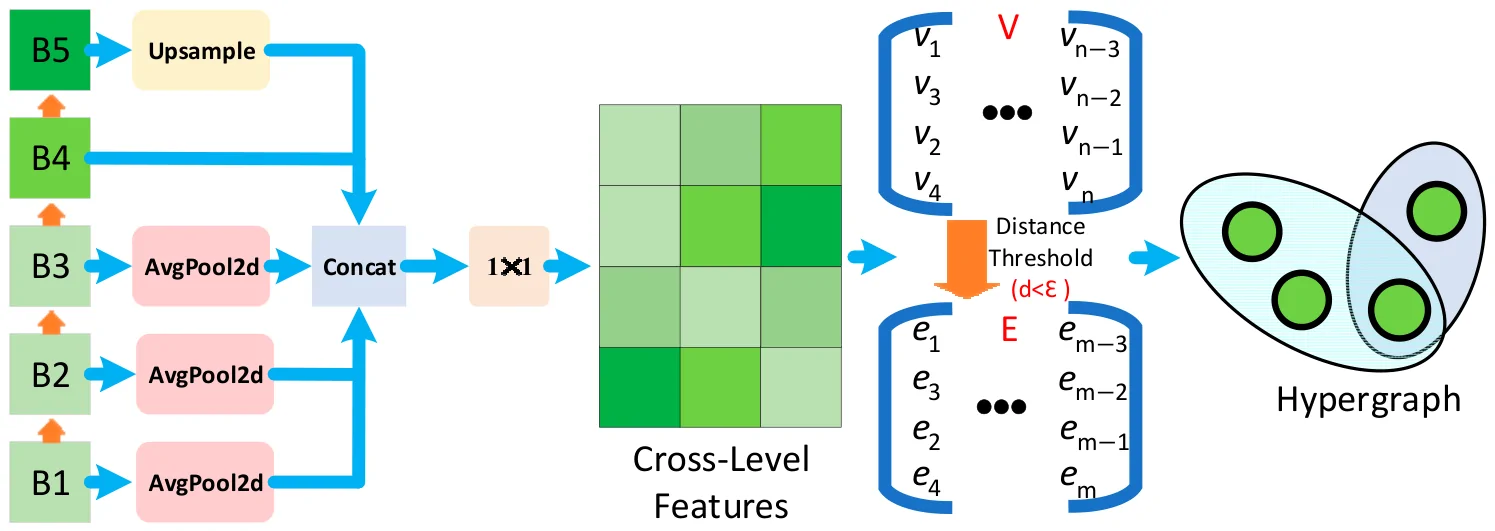
Multi-Scale hypergraph computation structure diagram.

On the one hand, given the vast differences in disease scale, feature representations at a single scale cannot adequately address detection requirements under extreme scales. On the other hand, since nutrient deficiency and viral diseases exhibit discrete distributions on leaves, conventional convolution cannot model the higher-order spatial correlations between scattered lesions. Therefore, this paper improves the Neck network by adopting a multiscale hypergraph aggregation structure [25] to fuse and enhance multi-level features, as shown in Figure 5.

First, we collect the semantic information from the multiscale features extracted by the backbone network. For the shallow high-resolution features B1, B2, and B3, large-scale average pooling is applied to extract global contextual information. For the deep low-resolution feature B5, upsampling is performed to restore spatial details. The multi-level features obtained after pooling and upsampling are concatenated along the channel dimension with the intermediate-layer feature B4 to construct rich global contextual information ranging from shallow texture to deep semantics. Next, a hypergraph computation module is introduced. The features output by the semantic collection layer undergo 1×1 convolution for dimensionality reduction to construct a node set $V=\{v_{1},v_{2},\ldots ,v_{n}\}$, where each node corresponds to a spatial location in the feature map. A filtering strategy is then used to construct a hyperedge set $E=\{e_{1},e_{2},\ldots ,e_{m}\}$. Specifically, the K-nearest neighbor strategy is first applied to select the K most similar nodes for each node. Then a Euclidean distance threshold ε, which is adaptively set based on the statistical distribution of pairwise distances, is used to filter out node pairs whose distances exceed ε. Specifically, the value of K in the K-nearest neighbor strategy, together with the distance threshold ε, is determined empirically through validation experiments, where ε is defined as the 70th percentile of pairwise Euclidean distances in feature space. In this way, each hyperedge connects multiple disease nodes that are spatially adjacent or feature-similar, thereby encoding the spatial distribution information of dispersed diseases into a hypergraph structure.

Formally, the hypergraph is represented by an incidence matrix $H\in R^{\left|V\right|\times \left|E\right|}$, where $H_{i,j}=1$ if node $v_{i}$ belongs to hyperedge $e_{j}$ and 0 otherwise. The connectivity of the hypergraph is further characterized by the hyperedge degree matrix $D_{e}=diag(\left|e_{j}\right|)$ and the node degree matrix $D_{v}=diag(\sum_{j=1}^{\left|E\right|}H_{i,j})$. Hypergraph convolution transmits information through the set of hyperedges according to the following layer-wise propagation rule, as shown in Equation (7) below:

(7) $$X^{\left(l+1\right)}=\sigma (D_{v}^{-1/2}HD_{e}^{-1}H^{T}D_{v}^{-1/2}X^{\left(l\right)}A_{w}^{\left(l\right)})$$

where $X^{\left(l\right)}$ denotes the node feature matrix at layer l, $A_{w}^{\left(l\right)}$ is the learnable weight matrix, and σ is the activation function. This propagation rule enables each node to aggregate information from all nodes within the same hyperedge, which is equivalent to a form of information diffusion governed by the hypergraph Laplacian. This facilitates the learning of latent associations among dispersed diseases and ultimately generates hypergraph node-enhanced features $F_{hyper}$. This endows the model with the ability to comprehensively distinguish between dispersed diseases, addressing the challenge of establishing spatial associations between nutrient deficiency disorders and viral diseases.

Finally, MANet further fuses multiscale information to balance the details of small-scale lesions with the global semantics of large-scale necrotic patches. It employs three parallel $Conv_{b}$ branches for adaptive aggregation: 1×1 convolution preserves features at the original scale, 3×3 hole convolution captures mid-range context, and 5×5 deep separable convolution obtains large-scale semantic information. After the outputs of the three branches are fused via attention-weighted averaging, residual connections are used to enhance the expression of the original feature $F_{original}$. The computational process is shown in Equation (8) below:


(8) $$F_{multi}=F_{original}+\sum_{b=1}^{3}\alpha_{b}\cdot Conv_{b}(F_{hyper})$$

Here, $\alpha_{b}$ represents the learnable fusion weight normalized by softmax function. MANet ensures that hypergraph node features are fully enhanced across different receptive fields, enabling a refined representation of multiscale features.

## 3. Experimental Environment

### 3.1. Experimental Setup

All models in this paper were trained and tested on AutoDL cloud servers under a standardized hardware and software environment to ensure the fairness and reproducibility of the experimental results. In terms of hardware configuration, the CPU used was an Intel Xeon Platinum 8481C, and the GPU used was an NVIDIA RTX 4090D (24 GB), 80 GB RAM. Regarding the software environment, the operating system was Ubuntu 22.04, and the deep learning framework was Python 3.10 and PyTorch 2.0.0. All models were trained and evaluated under this configuration with input images standardized to a size of 640 × 640 pixels. The experimental configuration is shown in Table 1.

### 3.2. Evaluation Metrics

To analyze the model’s effectiveness in detecting leaf diseases in tomatoes, Precision (P), Recall (R), Mean Average Precision (mAP), Parameters, and GFLOPs were selected as the primary metrics for evaluating model performance. The number of parameters is calculated based on the total trainable weights and biases in the final complete model. GFLOPs are calculated for a single forward pass with an input resolution of 640 × 640, following the standard computation rules for convolutional and fully connected layers. GFLOPs include all computation operations in the network, including hypergraph convolutions and sparse self-attention operations, so the comparison with other models’ complexity is fair and comprehensive.

Precision refers to the proportion of true positive samples correctly identified as positive out of the total number of positive samples predicted by the model. Precision reflects the model’s accuracy in predicting positive samples, as shown in Equation (9):


(9) $$Precision=\frac{N_{TP}}{N_{TP}+N_{FP}}$$

Recall refers to the proportion of positive samples that were correctly identified as positive out of the total number of positive samples. It reflects the model’s sensitivity in identifying positive samples. Recall is calculated as shown in Equation (10):


(10) $$Recall=\frac{N_{TP}}{N_{TP}+N_{FN}}$$

In the formula, $N_{TP}$ represents the number of positive samples correctly predicted by the model. $N_{FP}$ represents the number of positive samples incorrectly predicted by the model. $N_{FN}$ represents the number of positive targets that are missed by the model.

IoU is the ratio of the intersection area to the union area between the prediction bounding box (Pb) and the ground truth (Gt). When the IoU value between the prediction box output by the detection model and the ground truth box exceeds a specified threshold, the model’s predicted box is considered correct by default, as shown in Equation (11):


(11) $$IoU=\frac{Pb\cap Gt}{Pb\cup Gt}$$

AP is the area under the precision-recall curve for a single class. mAP is the average of the average precisions across *n* classes, and a higher value indicates better model performance. The formula is shown in Equation (12):


(12) $$mAP=\frac{1}{n}\sum_{i=1}^{n}\int_{0}^{1}Precision(Recall)d(Recall)$$

mAP50 is the mean average precision at an IoU threshold of 0.5; mAP75 is the mean average precision at an IoU threshold of 0.75; mAP50–95 is the mean average precision calculated from IoU thresholds ranging from 0.5 to 0.95 in steps of 0.05, which can more comprehensively evaluate the model’s performance across different IoU thresholds.

## 4. Results Analysis

### 4.1. Comparative Experiment

#### 4.1.1. Comparative Analysis of Different Disease Detections

To thoroughly evaluate TD-YOLO’s ability to detect different types of tomato leaf diseases, we compared the detection performance of the baseline model YOLOv11n and TD-YOLO across disease categories on the Tomato-Village-Diseases dataset. The experimental results are shown in Table 2. These results comprehensively assess the models’ ability to distinguish between physiological and viral diseases and clearly demonstrate the positive impact of the improvements on detection performance.

In terms of overall performance, TD-YOLO achieves significantly higher average accuracy than the baseline model across all categories. The mAP50 increases from 85.4% to 92.5%, with an absolute improvement of 7.1 percentage points. The mAP75 increases from 65.1% to 77.1%, with a improvement of 12.0 percentage points. This indicates that the improved model not only demonstrates a clear advantage in detection rate but also shows significant enhancement in bounding box localization accuracy. In particular, the substantial improvement in mAP75 indicates that the improved model can maintain high localization accuracy even at high IoU thresholds, which is especially important for accurately distinguishing between diseases with highly overlapping symptom areas, such as nitrogen deficiency and potassium deficiency.

For the healthy category, TD-YOLO achieves an mAP50 of 91.4%, which is 7.0 percentage points higher than the baseline of 84.4%. Meanwhile, the recall increases from 77.7% to 85.7%, with an improvement of 8.0 percentage points. This improvement is attributed to the C2TSSA attention mechanism’s effective suppression of background interference, which enables the model to more accurately distinguish between healthy and diseased leaves and reduces the false positive rate.

For the nitrogen-deficiency category, TD-YOLO achieves an mAP50 of 93.0%, which is 5.5 percentage points higher than the baseline of 87.5%. Its mAP75 reaches 79.7%, with an improvement of 11.4% over the baseline. The precision and recall attain 92.8% and 86.4%, increasing by 7.8 and 4.5 percentage points compared with the baseline, respectively. Nitrogen deficiency typically manifests as gradually changing colored patches with blurred boundaries, making detection challenging. The improved model uses RetC3k2 to enhance the shallow network’s ability to perceive subtle color changes, while the HyperConv module in the multiscale supergraph aggregation structure can model higher-order correlations between scattered yellowing areas, thereby significantly improving the detection accuracy for nitrogen deficiency.

For the potassium-deficiency category, TD-YOLO achieves an mAP50 of 93.2%, which is 8.0 percentage points higher than the baseline of 85.2%. The recall increases from 80.9% to 88.5%, with an improvement of 7.6 percentage points. Further analysis shows that the improved model obtains an mAP75 of 80.1% for potassium-deficiency, which is notably higher than the 79.7% for nitrogen-deficiency. This is because the leaf margin scorch characteristic of potassium deficiency has clearer boundaries, although the distribution of leaf margin scorch and yellowing symptoms is relatively scattered. Consequently, the combination of the leaf margin detail features retained by GDown and C2TSSA’s precise localization capability enables the model to accurately capture the contours of necrotic areas along the leaf margins.

For the spotted wilt virus category, TD-YOLO achieves an mAP50 of 92.3%, which is 8.0 percentage points higher than the baseline of 84.3%. Its mAP75 increases from 73.5% to 80.9%, with an improvement of 7.4 percentage points. The recall rises from 81.4% to 87.6%, gaining 6.2 percentage points. Spotted wilt virus manifests as annular patterns and necrotic spots on leaves, with complex and diverse symptom morphologies. In the multi-scale supergraph aggregation structure, the HyperConv module enhances the model’s overall ability to distinguish dense lesions by modeling the spatial distribution relationships among diseases, while the MANet network balances feature extraction from both the overall contours of annular spots and local necrotic points, significantly improving the detection accuracy of viral diseases.

Overall, TD-YOLO achieved consistent performance improvements across the four detection tasks—healthy, nitrogen deficiency, potassium deficiency, and spotted wilt virus, which fully validates the effectiveness of the proposed improvement strategy for detecting different types of tomato leaf diseases.

#### 4.1.2. Comparative Analysis of Different Detection Models

To verify the superiority of the TD-YOLO model in the tomato disease detection task, this paper compares its performance with that of other object detection models on the Tomato-Village-Diseases training dataset, including other models in the YOLO series and the classic detection model RT-DETR-R18. The results of the comparative experiments are shown in Table 3.

Experimental data show that, in terms of detection accuracy, the TD-YOLO model proposed in this paper achieves the best performance across all accuracy metrics. For the mAP50 metric, TD-YOLO reaches 92.5%, representing a 7.1% improvement over YOLOv11 and a 5.2% improvement over RT-DETR-R18. On the more stringent mAP50–95 metric, TD-YOLO achieved 68.3%, representing a 9.1% improvement over YOLOv13 and a 10.9% improvement over YOLOv11. This indicates that the model not only accurately detects disease targets but also achieves higher overlap between predicted and ground-truth bounding boxes, resulting in superior localization accuracy. In terms of precision and recall, TD-YOLO achieved 92.4% and 87.1%, respectively, representing a 6.1% and 6.6% improvement over the baseline YOLOv11 in both metrics. This indicates that the improved model effectively reduces both false positives and false negatives, which attributes to the C2TSSA mechanism’s selective focus on key diseases and the hypergraph computation’s ability to model higher-order associations among dispersed diseases.

In terms of lightweight performance, TD-YOLO also demonstrates significant advantages. The model has approximately 2.21M parameters, representing a reduction of about 88.9% compared to RT-DETR-R18’s 19.88M and a 14.6% reduction compared to YOLOv11’s 2.58M. Its computational complexity is only 6.8 GFLOPs, representing a 88.1% reduction compared to RT-DETR-R18. This demonstrates that through the synergistic design of RetC3k2 and C2TSSA, the model achieves effective lightweighting while maintaining high detection accuracy. Overall, TD-YOLO achieves an optimal balance between accuracy and efficiency, fully validating the effectiveness and superiority of the improvement strategies proposed in this paper, including the C2TSSA mechanism and multiscale supergraph aggregation in the complex field detection task of tomato leaf diseases.

#### 4.1.3. Model Adaptability Detection Comparison Analysis

To further validate the adaptability of the TD-YOLO model across different tomato leaf disease datasets, this study retrained the Tomato-Leaf-2-v1 dataset under the same environmental conditions and using the same parameter settings. The comparative experiments were repeated multiple times to ensure the reliability of the experimental data, and the results are shown in Table 4. The Tomato-Leaf-2-v1 dataset features a different distribution of disease categories and image acquisition conditions compared to the Tomato-Village-Diseases dataset, enabling an effective evaluation of the model’s adaptability across different data domains and disease categories.

In terms of detection accuracy, TD-YOLO achieved the best performance across all key metrics. Its mAP50 reached 81.0%, representing a 2.5% improvement over YOLOv13’s 78.5% and a 2.6% improvement over YOLOv11’s 78.4%. On the more stringent mAP75 metric, TD-YOLO achieved 48.9%, a 1.3% improvement over YOLOv11. The model achieves an mAP50–95 of 50.0%, which outperforms YOLOv13 by 0.7 percentage points, where the baseline performance of YOLOv13 is 49.3%. Precision and recall reached 79.1% and 75.2%, respectively, representing improvements of 1.8% and 2.5% over YOLOv11, indicating that the improved model can maintain low false negative and false positive rates across different data domains. The hypergraph computation within the HyperConv enables promising transferability for modelling high-order disease correlations among diverse disease categories, while the MANet multiscale aggregation network effectively mitigates detection challenges caused by variations in disease scale across different datasets. From a model lightweighting perspective, the parameter count of TD-YOLO is around 2.21M, which is reduced by 14.3% relative to YOLOv11 with 2.58M parameters. Its computational cost is 6.8 GFLOPs, on par with YOLOv8n and slightly higher than some lightweight models, but it achieves a significant improvement in accuracy with only a minimal increase in computational overhead. It is worth noting that TD-YOLO not only has fewer parameters than YOLOv8n but also achieves a 2.0% higher mAP50, fully demonstrating the architectural efficiency advantage of the RetC3k2 and C2TSSA collaborative design.

Based on the experimental results from the Tomato-Village-Diseases and Tomato-Leaf-2-v1 datasets, TD-YOLO consistently outperformed other models across different data distributions and disease categories. While maintaining a lightweight model, TD-YOLO demonstrates excellent adaptability capabilities and has the potential for applications in real-world and variable agricultural scenarios.

### 4.2. Ablation Experiment

This paper validates the effectiveness of the proposed improvements through ablation experiments. The experimental results are shown in Table 5, which presents 15 sets of ablation experiments. A, B, C, and D represent C2TSSA, RetC3k2, GDown, and Neck, respectively. The checkmark √ in Table 5 indicates that the corresponding ablation model contains the module. Here, Neck represents a multiscale hypergraph aggregation structure. This structure consists of three tightly coupled parts: the multiscale semantic aggregation layer provides cross-level context, the HyperConv modules model higher-order relationships among scattered lesions, and the MANet network performs multiscale aggregation on the hypergraph-enhanced features. Together, they form a progressive information flow, and missing any part would block the input dependencies for subsequent modules. Therefore, the Neck is evaluated as a complete module D to reflect its full functionality. By progressively applying each improvement strategy A, B, C, and D into the baseline model, we demonstrate varying degrees of improvement in detection performance.

The experimental results show that, in terms of the performance gains from introducing individual modules, each improved module enhances the detection performance of the baseline model to varying degrees. In Experiment 2, after introducing only C2TSSA, the mAP50 reaches 88.0%, representing an improvement of 2.6% over the baseline, and the precision is increased to 91.2%. With the number of parameters remaining essentially unchanged, this indicates that the C2TSSA mechanism can effectively suppress background noise interference, enabling the model to predict tomato leaf disease regions with greater precision and to locate areas of general yellowing caused by nitrogen deficiency and spots caused by leaf spot disease more accurately. In Experiment 3, after introducing RetC3k2 alone, the recall rate reached 83.1%, representing an improvement of 2.6% over the baseline. This demonstrates that RetC3k2, through its block-based residual enhancement strategy, effectively enhances the model’s feature representation capabilities while reducing computational costs, yielding significant gains in the extraction of leaf margin yellowing features associated with potassium deficiency. In Experiment 4, the standalone introduction of GDown achieved an mAP50 of 89.3%, with the number of parameters reduced to approximately 2.21M and computational complexity reduced to 5.4 GFLOPs, thereby simultaneously optimizing accuracy and efficiency. The adaptive downsampling strategy retained more spatial details, facilitating the detection of tiny early-stage spots caused by leaf spot disease. In Experiment 5, after introducing Neck alone, mAP75 reached 67.3%, representing an improvement of 2.2% over the baseline, indicating that semantic aggregation and hypergraph computation enhanced the ability to precisely localize multiscale diseases.

A comparison of Experiments 11 through 15 revealed that different module combinations exhibited synergistic effects, achieving simultaneous optimization of detection accuracy and computational efficiency. In Experiment 12, after introducing RetC3k2 (B) based on the combination of C2TSSA (A) and GDown (C), the mAP50 reached 90.9% and the recall rate increased to 85.9%, representing improvements of 2.2% and 3.4% respectively compared to Experiment 7. This indicates that RetC3k2 (B) and GDown (C) effectively complement each other in feature extraction and resolution compression. Furthermore, compared to Experiment 15, introducing Neck (D) on top of Experiment 12 increased mAP50 to 92.5%, mAP75 increased from 75.1% to 77.1%, and mAP50–95 increased from 65.3% to 68.3%, with precision and recall improving by 1.4% and 1.2%, respectively. This indicates that Neck (D), with its multiscale hypergraph aggregation structure, can fully leverage the rich features extracted by the first three components to achieve high-order semantic relationship modeling and multiscale feature enhancement. Ablation experiments treat the Neck as an overall module D because its internal multiscale semantic aggregation layer, HyperConv, and MANet are highly input-dependent. Removing any single component would disrupt the complete information flow, making split ablation meaningless. Future work could try designing decoupled alternative structures to analyze the contribution of each submodule of the Neck in more detail while maintaining full functionality.

In summary, the ablation experiment results fully validate the effectiveness and practicality of each improvement strategy in the tasks of detecting nitrogen deficiency, potassium deficiency, and spotted wilt virus in tomato leaves. The combined use of the four modules produces significant synergy, enabling TD-YOLO to achieve optimal performance in the tomato leaf disease detection task.

### 4.3. Visual Experiment

**Figure 6:**
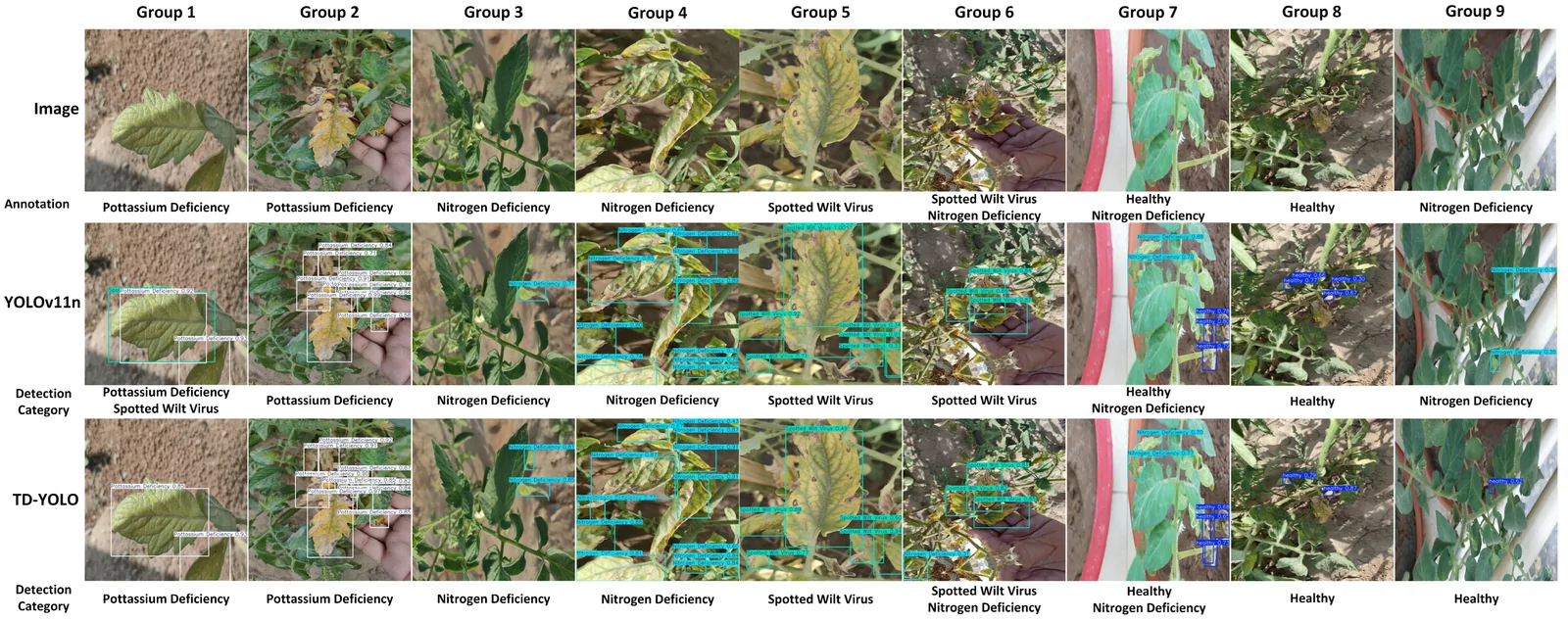
Tomato-Village-Diseases detection comparison chart.

To visually validate the detection advantages of TD-YOLO in complex field scenarios, images representing four categories from the Tomato-Village-Diseases test set were selected for a visual comparison analysis between YOLOv11n and TD-YOLO. The detection results are shown in Figure 6. From the perspective of overall detection performance, although YOLOv11n can locate most disease targets, it still suffers from issues such as missed detections, false positives, and low confidence scores when dealing with subtle early symptoms of nutrient deficiency, minute lesions of leaf spot disease, and interference from healthy leaf backgrounds. From the comparison in groups 1–4 in the figure, for nutrient deficiency detection, nitrogen deficiency manifests as uniform yellowing of leaves, while potassium deficiency manifests as a gradual browning of leaf margins. Both of which exhibit subtle early symptoms that are barely distinguishable from healthy leaves. YOLOv11n cannot detect such low-contrast symptoms, whereas TD-YOLO can accurately detect leaves with early-stage nutrient deficiency by enhancing the shallow network’s sensitivity to subtle color changes through the RetC3k2 module. From the comparison in groups 5–6 in the figure, for spotted wilt virus detection, early-stage lesions are small and scattered. YOLOv11n suffers from insufficient recall for small lesions and is prone to duplicate detections, whereas TD-YOLO can comprehensively detect scattered small lesions and eliminate redundant detection boxes, while improving confidence scores by effectively preserving detailed information through the GDown module. From the comparison in groups 7–8 in the figure, for healthy leaves detection, YOLOv11n tends to misclassify leaf veins or shadows as diseased regions. By selectively focusing on critical lesion tokens via the sparse self-attention mechanism of C2TSSA, TD-YOLO effectively suppresses background interference and achieves a notable reduction in false-positive detections. Group 9 in the figure shows a typical failure case where the TD-YOLO model mistakenly classified nitrogen-deficiency as healthy. This mainly happened because leaf occlusion and uneven lighting further weakened the prominent features of the disease spots, making their saliency score fall below the screening threshold, which ultimately led to misdetection. This failure case shows that TD-YOLO still has some limitations in fully detecting nitrogen deficiency under conditions with severe occlusion and uneven lighting, and it also points to ways to improve the sparse screening strategy.

**Figure 7:**
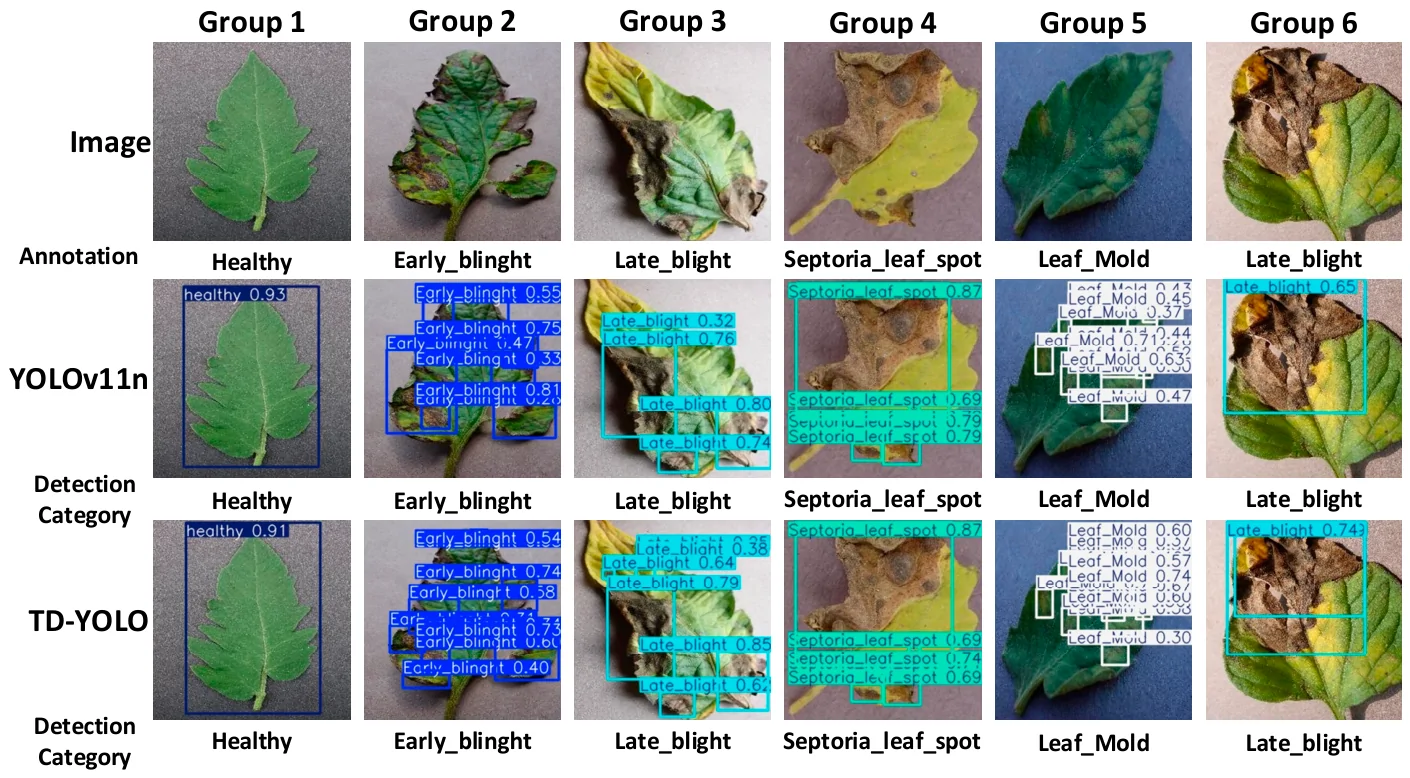
Tomato-Leaf-2-v1 detection comparison chart.

To verify the adaptability of TD-YOLO across different data domains and disease categories, a visual comparison analysis was conducted between YOLOv11n and TD-YOLO on the Tomato-Leaf-2-v1 dataset. The detection results are shown in Figure 7. Comparing groups 1–5 in the figure overall shows that YOLOv11n has issues with low confidence and inaccurate bounding box positioning for certain disease targets. This is particularly evident in multi-class scenarios where early_blight and late_blight coexist, where false negatives and class confusion are common. In contrast, TD-YOLO maintains high detection confidence and more precise bounding box localization across all disease categories. Its detection boxes align more closely with the actual contours of the diseases, resulting in a significant reduction in missed and false detections. When comparing groups 1–2 in the figure for distinguishing healthy leaves from early_blight, YOLOv11n notably fails to detect early_blight cases with subtle symptoms, whereas TD-YOLO can accurately identify these easily overlooked early symptoms owing to the enhanced detection capability for superficial lesions delivered by the RetC3k2 module. When comparing groups 3–5 in the figure for distinguishing late_blight, septoria_leaf_spot, and leaf_mold, YOLOv11n’s detection boxes exhibited positioning errors and failed to fully cover the affected areas. TD-YOLO achieves more complete and precise detection box localization by modeling the higher-order associations of scattered lesions through its hypergraph computation module. Group 6 in the figure presents a representative failure case, where the baseline model generates an incomplete bounding box that fails to cover the entire necrotic region, which can be attributed to the loss of boundary details of large and irregular lesions during repeated downsampling in the backbone network. The results demonstrate that TD-YOLO also exhibits excellent detection performance and robustness in detecting tomato leaf diseases across different regions and categories, validating its adaptability across various datasets and disease types.

## 5. Conclusions

To address the challenges of diverse lesion scales, spatially scattered lesion distribution and high computational cost of existing models for tomato leaf disease detection, this paper presents a lightweight detection model, TD-YOLO. This model incorporates the RetC3k2 module into the backbone network to enhance the ability of shallow layers to detect faint disease symptoms, employs the C2TSSA mechanism to filter out key diseases while suppressing background noise, and designs the GDown adaptive downsampling module to preserve detailed information while reducing resolution. In the Neck section, a multiscale hypergraph aggregation structure is constructed by integrating a multiscale semantic aggregation layer, HyperConv modules, and a MANet network. By modeling hyperedges to decompose higher-order correlations among diseases, the model achieves a refined representation of multiscale features. Experimental results show that this model achieves an mAP50 of 92.5% on the Tomato-Village-Diseases dataset, outperforming the baseline YOLOv11 by 7.1 percentage points, with only 2.21M parameters and 6.8 GFLOPs computational cost. On the Tomato-Leaf-2-v1 dataset, its mAP50 reaches 81.0%, surpassing the comparative models and verifying its favourable adaptability. This method significantly improves detection accuracy while maintaining a lightweight model, providing an efficient and feasible technical solution for the precise detection of tomato leaf diseases.

In future work, we will extend the proposed model to multi-crop disease detection tasks. By introducing semi-supervised learning and domain adaptation methods, we aim to reduce the model’s dependence on data annotations for new scenarios.

## Figures and Tables

**Table 1:** Experimental setup.

Parameter	Epochs	Batch Size	Workers	Optimizer	Lr0	Momentum	Weigh Decay
Value	150	16	8	SGD	0.01	0.937	0.0005

**Table 2:** Comparison of different disease detections.

Type	YOLOv11n					TD-YOLO				
	P	R	mAP50	mAP75	mAP50–95	P	R	mAP50	mAP75	mAP50–95
ALL	0.863	0.805	0.854	0.651	0.574	0.924	0.871	0.925	0.771	0.683
Healthy	0.847	0.777	0.844	0.538	0.507	0.905	0.857	0.914	0.676	0.605
Nitrogen Deficiency	0.85	0.819	0.875	0.683	0.604	0.928	0.864	0.93	0.797	0.708
Potassium Deficiency	0.878	0.809	0.852	0.648	0.567	0.936	0.885	0.932	0.801	0.704
Spotted Wilt Virus	0.878	0.814	0.843	0.735	0.619	0.926	0.876	0.923	0.809	0.717

**Table 3:** Comparative experiments on the Tomato-Village-Diseases dataset.

Model	P	R	mAP50	mAP75	mAP50–95	Parameters	GFLOPs
RT-DETR-R18	0.908	0.791	0.873	0.703	0.632	19,876,896	57.0
YOLOv5n [26]	0.845	0.754	0.829	0.615	0.539	2,182,444	5.8
YOLOv6n [27]	0.809	0.685	0.767	0.577	0.512	4,155,420	11.5
YOLOv8n [28]	0.862	0.781	0.848	0.661	0.579	2,685,148	6.8
YOLOv9t	0.862	0.752	0.835	0.631	0.555	1,730,604	6.4
YOLOv10n [29]	0.826	0.74	0.823	0.615	0.527	2,265,948	6.5
YOLOv11n	0.863	0.805	0.854	0.651	0.574	2,582,932	6.3
YOLOv12 [30]	0.793	0.722	0.776	0.564	0.494	2,509,124	5.8
YOLOv13 [31]	0.863	0.797	0.864	0.676	0.592	2,448,675	6.1
TD-YOLO	0.924	0.871	0.925	0.771	0.683	2,207,854	6.8

**Table 4:** Comparative experiments on the Tomato-Leaf-2-v1 dataset.

Model	P	R	mAP50	mAP75	mAP50–95	Parameters	GFLOPs
YOLOv5n	0.766	0.722	0.78	0.46	0.475	2,182,639	5.8
YOLOv6n	0.752	0.697	0.753	0.447	0.467	4,155,519	11.5
YOLOv8n	0.774	0.732	0.79	0.47	0.488	2,685,343	6.8
YOLOv9t	0.757	0.725	0.781	0.47	0.488	1,730,799	6.4
YOLOv10n	0.753	0.717	0.776	0.466	0.473	2,266,143	6.5
YOLOv11n	0.773	0.727	0.784	0.476	0.488	2,583,127	6.3
YOLOv12	0.758	0.718	0.774	0.464	0.477	2,509,319	5.8
YOLOv13	0.773	0.722	0.785	0.475	0.493	2,448,870	6.1
TD-YOLO	0.791	0.752	0.81	0.489	0.5	2,208,049	6.8

**Table 5:** Ablation experiments.

Method	A	B	C	D	P	R	mAP50	mAP75	mAP50–95	Parameters	GFLOPs
1					0.863	0.805	0.854	0.651	0.574	2,582,932	6.3
2	√				0.912	0.792	0.88	0.689	0.606	2,565,014	6.3
3		√			0.885	0.831	0.888	0.69	0.601	2,582,876	6.3
4			√		0.909	0.819	0.893	0.702	0.615	2,212,180	5.4
5				√	0.863	0.816	0.871	0.673	0.597	2,544,084	7.1
6	√	√			0.915	0.812	0.879	0.687	0.603	2,564,958	6.3
7	√		√		0.909	0.825	0.887	0.718	0.622	2,194,262	5.4
8	√			√	0.886	0.824	0.879	0.695	0.607	2,526,166	7.0
9		√	√		0.913	0.857	0.914	0.754	0.664	2,264,620	6.1
10		√		√	0.901	0.825	0.893	0.72	0.638	2,596,524	7.7
11			√	√	0.912	0.836	0.898	0.721	0.634	2,173,332	6.1
12	√	√	√		0.91	0.859	0.909	0.751	0.653	2,246,702	6.0
13	√	√		√	0.892	0.838	0.891	0.733	0.641	2,578,606	7.7
14		√	√	√	0.922	0.858	0.917	0.757	0.673	2,225,772	6.8
15	√	√	√	√	0.924	0.871	0.925	0.771	0.683	2,207,854	6.8

## Data Availability

The data presented in this study are available in publicly accessible repositories. The Tomato-Village-Diseases dataset used in this study is publicly available at https://universe.roboflow.com/tomatodiseases/tomato-village-diseases (accessed on 28 September 2026). The Tomato-Leaf-2 dataset used in this study is publicly available at https://universe.roboflow.com/tomatoleaf-lprro/tomato-leaf-2/dataset/1 (accessed on 28 September 2026).

## References

[1] Savary S., Willocquet L., Pethybridge S.J., Esker P., McRoberts N., Nelson A. (2019). The global burden of pathogens and pests on major food crops. Nat. Ecol. Evol..

[2] Kotwal J., Kashyap R., Pathan S. (2023). Agricultural plant diseases identification: From traditional approach to deep learning. Mater. Today Proc..

[3] Shafik W., Tufail A., Namoun A., De Silva L.C., Apong R.A.A.H.M. (2023). A systematic literature review on plant disease detection: Motivations, classification techniques, datasets, challenges, and future trends. IEEE Access.

[4] Shoaib M., Sadeghi-Niaraki A., Ali F., Hussain I., Khalid S. (2025). Leveraging deep learning for plant disease and pest detection: A comprehensive review and future directions. Front. Plant Sci..

[5] Amulothu D.V.R.T., Rodge R.R., Hasan W., Gupta S. (2024). Machine learning for pest and disease detection in crops. Agriculture 4.0.

[6] Girshick R., Donahue J., Darrell T., Malik J. Rich feature hierarchies for accurate object detection and semantic segmentation. Proceedings of the IEEE Conference on Computer Vision and Pattern Recognition.

[7] Ren S., He K., Girshick R., Sun J. (2016). Faster R-CNN: Towards real-time object detection with region proposal networks. IEEE Trans. Pattern Anal. Mach. Intell..

[8] Redmon J., Divvala S., Girshick R., Farhadi A. You only look once: Unified, real-time object detection. Proceedings of the IEEE Conference on Computer Vision and Pattern Recognition.

[9] Khanam R., Hussain M. (2024). Yolov11: An overview of the key architectural enhancements. arXiv.

[10] Arwidiyarti D. (2025). Single shot multibox detector (SSD) in object detection: A review. IJACI Int. J. Adv. Comput. Inform..

[11] Zhao Y., Lv W., Xu S., Wei J., Wang G., Dang Q., Liu Y., Chen J. Detrs beat yolos on real-time object detection. Proceedings of the 2024 IEEE/CVF Conference on Computer Vision and Pattern Recognition (CVPR).

[12] Abulizi A., Ye J., Musideke M., Zhang G., Su Y., Zhang Y., Abudukelimu H. (2026). FNE-RTDETR: A lightweight end-to-end model for small-area tomato leaf disease detection and fine-grained classification. Neural Netw..

[13] Bian S., Su S., Zhou J., Yi C., Huang F. (2025). Multi-scale feature fusion based RT-DETR for tomato leaf disease detection in complex backgrounds. Sensors.

[14] Xing P., Tang H., Tang J., Li Z. (2024). ADPS: Asymmetric distillation postsegmentation for image anomaly detection. IEEE Trans. Neural Netw. Learn. Syst..

[15] Tang H., Li Z., Zhang D., He S., Tang J. (2024). Divide-and-conquer: Confluent triple-flow network for RGB-T salient object detection. IEEE Trans. Pattern Anal. Mach. Intell..

[16] Ramos L.T., Sappa A.D. (2025). A comprehensive analysis of YOLO architectures for tomato leaf disease identification. Sci. Rep..

[17] Meng X., Chen C., Dong W., Wang K. (2025). Tomato Leaf Disease Detection Method Based on Multi-Scale Feature Fusion. Plants.

[18] Tang X., Sun Z., Yang L., Chen Q., Liu Z., Wang P., Zhang Y. (2025). YOLOv11-AIU: A lightweight detection model for the grading detection of early blight disease in tomatoes. Plant Methods.

[19] Wang M., Cai J., Liu W., Yang X., Zhang J., Zhou Q., Wang F., Zhang H., Liu T. (2026). YOLO-SPDNet: Multi-Scale Sequence and Attention-Based Tomato Leaf Disease Detection Model. Phyton.

[20] Han Y., Zhu Y., Hu T., Lan Y., Huang D., Zhao S. (2026). DFR-YOLOv12n: A Lightweight Detection Method for Tomato Leaf Diseases in Natural Environments via Detail-Preserving Downsampling, Feature Fusion Enhancement, and Regression Optimization. Horticulturae.

[21] Zarboubi M., Bellout A., Chabaa S., Dliou A. (2025). CustomBottleneck-VGGNet: Advanced tomato leaf disease identification for sustainable agriculture. Comput. Electron. Agric..

[22] Xu Y., Wu H., Liu Y., Zhang X. (2025). PCB electronic component soldering defect detection using YOLO11 improved by retention block and neck structure. Sensors.

[23] Wang C.Y., Yeh I.H., Liao M. (2024). Yolov9: Learning what you want to learn using programmable gradient information. Computer Vision—ECCV 2024, Proceedings of the 18th European Conference, Milan, Italy, 29 September–4 October 2024.

[24] Wu Z., Ding T., Lu Y., Pai D., Zhang J., Wang W., Haeffele B. Token Statistics Transformer: Linear-Time Attention via Variational Rate Reduction. Proceedings of the 13th International Conference on Learning Representations.

[25] Feng Y., Huang J., Du S., Ying S., Yong J.-H., Li Y., Ding G., Ji R., Gao Y. (2024). Hyper-yolo: When visual object detection meets hypergraph computation. IEEE Trans. Pattern Anal. Mach. Intell..

[26] Luo H., Wei J., Wang Y., Chen J., Li W. (2024). An improved lightweight object detection algorithm for YOLOv5. PeerJ Comput. Sci..

[27] Li C., Li L., Jiang H., Weng K., Geng Y., Li L., Ke Z., Li Q., Cheng M., Nie W. (2022). YOLOv6: A single-stage object detection framework for industrial applications. arXiv.

[28] Reis D., Kupec J., Hong J., Daoudi A. (2023). Real-time flying object detection with YOLOv8. arXiv.

[29] Wang A., Chen H., Liu L., Chen K., Lin Z., Han J., Ding G. (2024). Yolov10: Real-time end-to-end object detection. Advances in Neural Information Processing Systems 37, Proceedings of the 38th Conference on Neural Information Processing Systems (NeurIPS 2024).

[30] Tian Y., Ye Q., Doermann D. (2026). Yolov12: Attention-centric real-time object detectors. Advances in Neural Information Processing Systems 38, Proceedings of the 39th Conference on Neural Information Processing Systems (NeurIPS 2025).

[31] Lei M., Li S., Wu Y., Hu H., Zhou Y., Zheng X., Ding G., Du S., Wu Z., Gao Y. (2025). Yolov13: Real-time object detection with hypergraph-enhanced adaptive visual perception. arXiv.

